# Individualized Muscle-Tendon Assessment and Training

**DOI:** 10.3389/fphys.2020.00723

**Published:** 2020-06-26

**Authors:** Adamantios Arampatzis, Falk Mersmann, Sebastian Bohm

**Affiliations:** ^1^Department of Training and Movement Sciences, Humboldt-Universität zu Berlin, Berlin, Germany; ^2^Berlin School of Movement Science, Humboldt-Universität zu Berlin, Berlin, Germany

**Keywords:** diagnostic, ultrasound, mechanical properties, performance, individualization

## Abstract

The interaction of muscle and tendon is of major importance for movement performance and a balanced development of muscle strength and tendon stiffness could protect athletes from overuse injury. However, muscle and tendon do not necessarily adapt in a uniform manner during a training process. The development of a diagnostic routine to assess both the strength capacity of muscle and the mechanical properties of tendons would enable the detection of muscle-tendon imbalances, indicate if the training should target muscle strength or tendon stiffness development and allow for the precise prescription of training loads to optimize tendon adaptation. This perspective article discusses a framework of individualized muscle-tendon assessment and training and outlines a methodological approach for the patellar tendon.

## Introduction

It has been long recognized that the functional properties of muscles are a crucial determinant of movement performance in both every day and athletic activities (Masterson, 1976; Delecluse, 1997). Therefore, their assessment, especially in terms of muscle strength, is now a standard diagnostic component when monitoring for example performance in sports (Smith et al., 2002; McMaster et al., 2014) or the recovery process in rehabilitation (Osternig, 1986). In comparison, we just recently began to understand how tendons influence muscle-tendon unit (MTU) functioning and performance (Kawakami and Fukunaga, 2006; Roberts, 2016). In the practical field of sports and rehabilitation, the assessment of tendon properties is until now mostly confined to medical imaging in the context of injuries (Robinson, 2009). In this article, we want to make an argument that a differentiated diagnostic of muscle functional and tendon mechanical properties could be a promising approach to individualize training loads. The approach would allow to specifically target muscle or tendon adaptation and facilitate a balanced development of the contractile and series elastic elements of the MTU. Developing effective strategies how to manipulate the interaction of muscle and tendon could make an important contribution for the development of physical performance as well as the prevention and rehabilitation of injuries.

Owing to systematic research endeavors of this century, it is now clearly established that human tendons can adapt to mechanical loading across the lifespan (Waugh et al., 2014; Bohm et al., 2015; McCrum et al., 2018). However, there is also evidence that the functional properties of muscles and the mechanical properties of tendons do not necessarily change in a similar manner during a training process (Mersmann et al., 2017a). For example, tendons do not adapt as quickly to mechanical loading as muscles due to a lower rate of tissue renewal (Heinemeier et al., 2013). Further, not all types of loading that increase muscle strength are effective in stimulating an increase of tendon stiffness, which is the resilience of the tendon according to its force-elongation relationship. For example, plyometric training and fatiguing training with moderate loads show clear effects on muscle strength and hypertrophy (Sáez-Sáez de Villarreal et al., 2010; Schoenfeld, 2013), yet lower, less consistent or even no effects on the stiffness of the tendon (Arampatzis et al., 2007a; Burgess et al., 2007; Kubo et al., 2007; Bohm et al., 2014). If an increase in the muscle’s capacity to generate force is not accompanied by an adequate increase in tendon stiffness, the tendon is subjected to higher levels of strain during a muscle contraction at a given relative intensity. As the ultimate strain of tendons is remarkably constant (LaCroix et al., 2013), an increase of tendon operating strain during muscle contraction implies an increase of the mechanical demand placed upon the tendon.

An imbalanced development of muscle and tendon has implications for (a) movement performance, (b) the risk of injury and (c) the prescription of training loads. Though movement performance is certainly a complex interplay of musculoskeletal (Cormie et al., 2011; Suchomel et al., 2016), neural (Yarrow et al., 2009) and psychological factors (Raglin, 2001), the interaction of muscle and tendon is an integral part with regard to how we produce forces to move. Although on an individual level there is little information concerning muscle-tendon imbalances and specific competitive performance, there are reports that for optimal muscle interaction, muscle strength and tendon stiffness need to be well matched (Lichtwark and Wilson, 2007; Orselli et al., 2017) and controlled via a finely tuned neural drive to the muscle (Sawicki et al., 2015). An imbalance in muscle and tendon adaptation might impair this interplay, which would reduce the efficiency of the musculotendinous energy exchange. Moreover, an increase in operating strain reduces the tendon safety factor (ratio of operating strain to ultimate strain) and may increase the risk of injury. The initial strain induced in a tendon at a given load determines the time to rupture during both static and cyclic loading (Wren et al., 2003). That is why strain-induced tissue damage is considered one of the major mechanical risk factors for the development of tendinopathy (Fredberg and Stengaard-Pedersen, 2008; Lavagnino et al., 2008; Wang et al., 2013). Finally, potential imbalances in muscle and tendon adaptation imply that the prescription of training loads for the tendon is not precise when it is based on the strength capacity of the muscle (e.g., setting the training intensity to a percentage of the one-repetition- or isometric maximum). An effective training stimulus for the tendon is expected at contraction-induced tendon strains of 4.5 to 6.5% (Arampatzis et al., 2007a, 2010; Bohm et al., 2014), which does not correspond to the same intensity of muscle contraction for each individual. Therefore, a differentiated diagnostic of muscle and tendon properties would open up opportunities to optimize loading during training and, thus, facilitate adaptation for the improvement of physical performance or the prevention and rehabilitation of overuse injuries.

## Framework of the Individualized Muscle-Tendon Assessment and Training

Tendons, as mostly collagenous structures, are not able to contribute to the active force generation of the muscle-tendon unit. However, due to their compliance, they can significantly affect muscle force production and, thus, are an important component of the human musculoskeletal system for effective locomotion (Roberts, 1997; Lai et al., 2014). Several studies in the last 10-15 years provided important information regarding the Achilles tendon and aponeurosis deformation during different tasks as for example for walking (Lichtwark et al., 2007; Lai et al., 2015), running (Lichtwark et al., 2007; Albracht and Arampatzis, 2013; Lai et al., 2018) and jumping (Kurokawa et al., 2003; Lichtwark and Wilson, 2005; Ishikawa and Komi, 2008). The reported maximum strains of the Achilles tendon during these activities were calculated from muscle fascicle behaviour and range between 4.3% during walking (Lichtwark et al., 2007) up to 9.0% strain in fast running (Lai et al., 2018). Furthermore, current studies investigating the function of the knee extensor MTU evidenced significant deformation of the quadriceps and patellar tendon during jumping (Nikolaidou et al., 2017), landing (Hollville et al., 2019), walking and running (Bohm et al., 2018). These findings demonstrate that a certain deformation of tendons is required during daily life and sport activities for an effective locomotion. This tendon deformation is important because it affects both the force-length-velocity and power-velocity potential of the muscle (Nikolaidou et al., 2017; Bohm et al., 2018, 2019) as well as strain energy storage and return within the MTU (Lichtwark and Wilson, 2005; Ishikawa and Komi, 2008; Lai et al., 2014). Consequently, the muscle has to be strong enough to appropriately deform the tendon and to use tendon elasticity for an efficient muscle-tendon interaction during movement. However, both too high and too low levels of habitual deformation may be associated with impairments of tendon structure. Though the exact ultimate strain of human tendons cannot be determined *in vivo*, it is clear that excessive tendon deformations increase the mechanical demand for the tendon, since *in vitro* data shows that ultimate tendon strain is remarkably constant (LaCroix et al., 2013). Therefore, high operating to ultimate strain ratios increase the risk of tissue failure (Wren et al., 2003). Wang et al. (2013) demonstrated that cyclic strains of 9.0% act degenerative on the tendon structure and weaken its structural integrity. However, the study also provided evidence that also too low deformations (≤ 3.0% strain) may induce catabolic signaling and matrix deterioration.

In a series of systematic intervention studies, we modulated tendon strain magnitude (3% and 4.5–6.5%), frequency (0.17 and 0.5 Hz), strain rate (modulated via time to peak force of ∼130 and ∼380 ms) and duration (1 s, 3 s and 12 s) while controlling for overall loading volume. We found that cyclic loading of the tendon with strain values between 4.5 and 6.5% and a duration of 3 s per repetition (applied with the low frequency and strain rate) was the most effective mechanical stimulus for the improvement of human tendon mechanical properties *in vivo* (Arampatzis et al., 2007a, 2010; Bohm et al., 2014). Tendon exercise loading with strain values of ∼3.0% on the other hand did not improve tendon mechanical properties (Arampatzis et al., 2007a, 2010). In accordance with our findings, also other authors conclude from their recent experimental results (Wang et al., 2013) or literature review (Pizzolato et al., 2018; Docking and Cook, 2019) that there is an optimal range of tendon strain during exercise for triggering tendon adaptation and promoting its mechanical and morphological properties. The deformation of the tendon during exercise can be regulated by the muscle force generation and strains of 4.5 to 6.5% are usually achieved at about 90% of a voluntary maximum isometric contraction (MVC; Arampatzis et al., 2007a, 2010; Bohm et al., 2014). However, on the individual basis, this might not necessarily be the case.

**FIGURE 1:**
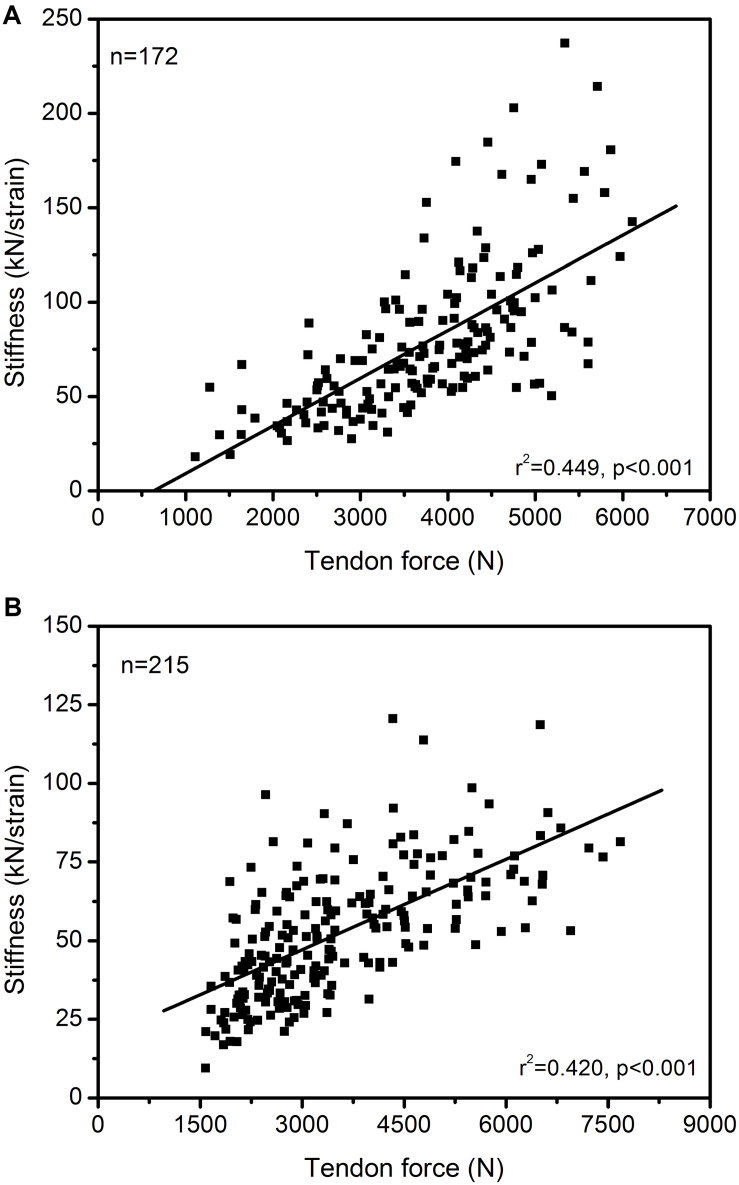
Association between *in vivo* Achilles **(A)** and patellar **(B)** tendon force and tendon stiffness (normalized to tendon rest length) in 172 and 215 athletes from different sports (endurance running, sprinting, ball sports, diverse) and untrained individuals, including data from adolescents and adults. The Pearson correlation coefficients and 95% confidence intervals [lower limit, upper limit] were 0.670 [0.578, 0.745] and 0.648 [0.563, 0.719] for the Achilles and patellar tendon, respectively. The presented data is from earlier studies of our group (A: Arampatzis et al., 2007a, b, 2010; Stafilidis and Arampatzis, 2007; Albracht and Arampatzis, 2013; B: Charcharis et al., 2019; Mersmann et al., 2019) as well as yet unpublished work (A: *n* = 26; B: *n* = 118).

The maximum muscle strength and tendon stiffness are the two parameters that regulate maximum strain of the tendon during muscle contractions. An imbalance between muscle strength and tendon stiffness can result in either too low or too high tendon strain during maximum contractions with potential negative consequences for both performance capabilities and tendon health (Mersmann et al., 2017a, 2019). In general, there is a strong association between muscle strength and tendon stiffness, at least in triceps surae and quadriceps MTUs. This has been reported for children (Waugh et al., 2012; Pentidis et al., 2019), adolescents (Charcharis et al., 2019; Mersmann et al., 2019), young (Arampatzis et al., 2007b; Epro et al., 2019) and old adults (Stenroth et al., 2012; Epro et al., 2017). Figure 1 shows the correlation of plantar flexor muscle strength with Achilles tendon stiffness and quadriceps muscle strength with patellar tendon stiffness in 172 and 215 athletes, respectively. The significant association between muscle strength and tendon stiffness in both MTUs support the idea that, in general, muscle strength and tendon stiffness show a coordinated adaptation and that individuals with higher muscle strength also have stiffer tendons. However, a significant relationship between muscle strength and tendon stiffness does not give evidence to a balanced adaptation within the MTU, because a high or low relationship does not provide any information concerning the margin of tolerated mechanical tendon loading during MVCs. There is experimental evidence of imbalances between muscle strength and tendon stiffness in competitive athletes from child- to adulthood due to different alterations of muscle and tendon properties, resulting in remarkably high or low tendon strain values (Mersmann et al., 2016; Charcharis et al., 2019; Pentidis et al., 2019). Those imbalances indicate the relevance of an individualized training control and regulation. If the maximum tendon strain during an MVC is too high (>9.0%), tendon stiffness seems too low compared to the strength capacity of the associated muscle and we would recommend a training that focusses on tendon adaptation (i.e., loading that causes 4.5 to 6.5% tendon strain in five sets of four repetitions with a loading-unloading duration of 3 s each and an inter-set rest of 2 min according to our recommendations; Mersmann et al., 2017a). If, on the other hand, the maximum strain is quite low (<4.5%), muscle strength seems too low compared to the stiffness of its tendon and a training that focused on muscle growth is indicated. Such scenarios can occur on an individual basis in athletes and need an individualized training regulation within the MTU. The strain levels suggested are not to be understood as cut-off criteria for injury prediction or fixed thresholds yet as transition bands into high or low levels of maximum strain. This information then can be used to individualize training, aiming to counteract muscle-tendon imbalances. In the authors’ view and considering the experimental data reviewed here, it is likely beneficial for performance capacity and injury risk if tendon stiffness is geared to muscle strength.

**FIGURE 2:**
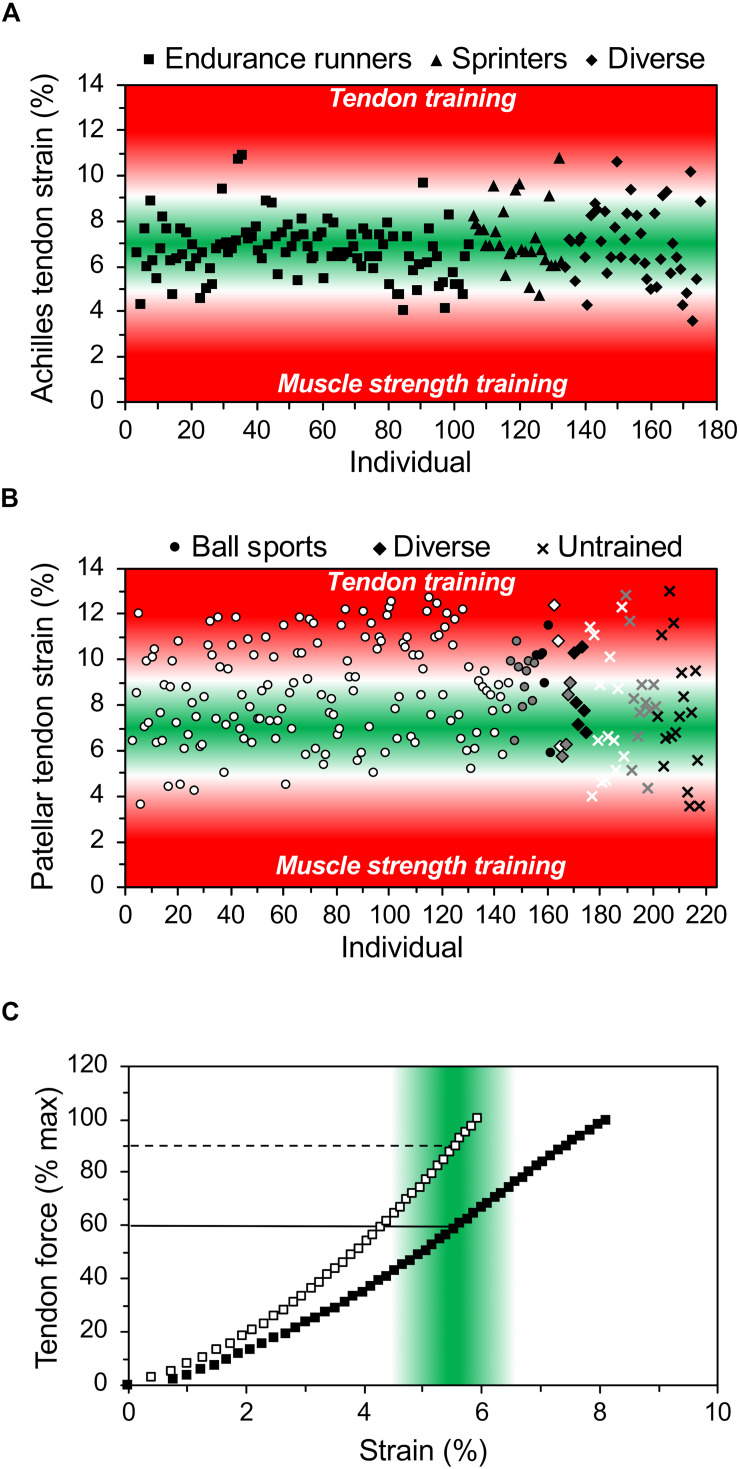
*In vivo* Achilles **(A)** and patellar **(B)** tendon strain during maximum voluntary isometric contractions in 172 and 215 athletes from different sports (endurance running, sprinting, ball sports, diverse) and untrained individuals from child- to adulthood (white: early adolescent [12–15 years], gray: late-adolescent [16–19 years], black: adults [≥20 years]). While low levels of tendon strain suggest that the athlete may focus on muscle strength development, high levels of strain indicate the need for specific tendon training for increasing its stiffness. **(C)** Illustrates the individual relationship of tendon force (in percent of maximum tendon force) and strain in two athletes. The green area indicates the range of strain where an optimal mechanical stimulation for training is expected and the horizontal lines show that the respective relative training intensity in terms of force exertion may differ substantially between individuals. The presented data is from earlier studies of our group (A: Arampatzis et al., 2007a, b, 2010; Stafilidis and Arampatzis, 2007; Albracht and Arampatzis, 2013; B: Charcharis et al., 2019; Mersmann et al., 2019) as well as yet unpublished work (A: *n* = 26; B: *n* = 118). Strain was extrapolated based on stiffness for the tendon forces during maximum voluntary isometric contractions in the respective optimum joint angle. For details on the respective methods see Arampatzis et al. (2007a) and Mersmann et al. (2016).

In our opinion, the assessment of the appropriate relationship between maximum muscle strength and tendon stiffness, using the maximum tendon strain during an MVC as diagnostic marker, is important for the training process. Imbalances between muscle strength and tendon stiffness can be identified in an early stage and customized decisions can be made for the training regulation of the individual athlete. Figures 2A,B shows the maximum strain values of the Achilles and patellar tendon of a high number of athletes during an MVC. It is visible that there are athletes who show either markedly high or low strain values and, thus, we would suggest personalized justification with focusing on muscle strength or tendon stiffness training, respectively. In athletes with maximum tendon strain higher than 11.0% the specific training to increase tendon stiffness seems crucial, while in others with strain values of 9.0 to 10.0% a slight correction in training content might suffice. There are also athletes that show maximum strain values <4.5%, which suggests that a customized training for muscle hypertrophy to increase muscle strength might be beneficial.

The relationship between muscle strength and tendon stiffness is further important for the definition of the optimal exercise intensity for tendon adaptation. It is well accepted that both muscle hypertrophy as well as muscle strength can be improved using low intensity exercise (e.g., 30% of one-repetition maximum) with high number of repetitions until fatigue (Mitchell et al., 2012). However, low intensity exercise does usually not initiate sufficient tendon strain to initiate adaptive changes of tendon properties (Bohm et al., 2015; Wiesinger et al., 2015). As mentioned above, the effective mechanical loading for tendon adaptation should cause tendon strains between 4.5 and 6.5%, which corresponds in average to a tendon loading of 90% MVC (Arampatzis et al., 2007a, 2010; Bohm et al., 2014). The individual and different relationship between muscle strength and tendon stiffness in athletes implies, however, that there can be substantial variations in terms of the percentage of the MVC at which the target levels of tendon strain for training are reached (Figure 2C). Therefore, the individual assessment of the MVC-strain relationship of the tendon is relevant for the definition of the optimal loading intensity, since it allows to individually fit the target strain (4.5–6.5%) to the MVC for a personalized tendon training.

It has to be mentioned that the origin of tendon pathology is multifactorial and currently there is not a clear factor or concert of factors that explain or precisely predict the occurrence of tendinopathy (Magnusson et al., 2010; Cook et al., 2016). The prevention of imbalances within the muscle-tendon unit might, however, reduce the risk of overload and, thus, a key risk factor of tendinopathy, while other risk factors related to genetics, age or recovery time and others certainly still contribute to tendon collagen turnover, pathology and function. Our proposed approach is intended to be used in the practical field to detect at an early stage if a tendon is in an unfavorable loading environment due to muscle-tendon imbalances. It is then possible to prescribe individualized training recommendations aiming to promote an efficient energy exchange between muscle and tendon and to counteract the potential development of overuse. Several additional methodologies including ultrasound tissue characterization (van Schie et al., 2010; Visnes et al., 2015), spatial frequency analysis of ultrasound images (Bashford et al., 2008; Mersmann et al., 2019), intra-tendinous motion (Couppé et al., 2020), tissue biopsy and microdialysis (Magnusson et al., 2010) can be used to improve our understanding of tendon pathogenesis and function.

## Potential Practical Implementation at the Example of the Patellar Tendon

Individualizing exercise prescriptions for muscle and tendon training requires an assessment of muscle strength and tendon mechanical properties. The measurement of a tendon force-elongation relationship *in vivo* is, however, associated with considerable methodological effort (Seynnes et al., 2015). But, allowing for some simplified assumptions, it seems possible to develop a diagnostic setup for the application in the field. First, as tendon force is approximately proportional to the generated joint moment during isometric contractions, the assessment of the tendon moment arm and calculation of tendon forces could be omitted. The relationship of externally measured moments or forces to the elongation of the tendon would therefore be representative of tendon stiffness. While interindividual comparisons of such a measure of tendon stiffness would be biased by differences in the tendon moment arm, longitudinal changes should be well represented as long as no major change of moment arm within individuals can be expected (i.e., in adults). Second, though there might be differences in antagonist coactivation between untrained and trained cohorts that affect the ratio of externally measured force or moment to the actual tendon force, after a few accustoming sessions no major changes in the relative contribution of the antagonist to the resultant joint moment are to be expected (Carolan and Cafarelli, 1992). Recently, we measured knee joint moments in 14 adolescent basketball athletes at four measurement time points of a competitive season and observed only marginal fluctuations of the antagonist moment of 2.5 ± 1.5%. Third, while the elaborate assessment of tendon cross-sectional area is necessary to understand the mechanisms of tendon adaptation in the scientific field, for monitoring training adaptations and prescribing exercise it seems sufficient to confine the outcome parameters to tendon stiffness or even only to tendon strain.

Tendon mechanical properties *in vivo* are measured during isometric contractions. For the assessment at the patellar tendon, we would suggest a seated position with the knee flexed to 90°, as in this position passive forces resolve tendon slack (yet not causing substantial elongation), which simplifies the measurement of tendon rest length and elongation, and the alignment of the force sensor with the force vector can be more easily controlled. It needs to be mentioned that a 90° knee joint angle not optimal for maximal moment generation and can result in lower tendon strain values, which needs to be kept in mind in their interpretation. After a standardized warm-up and a series of at least 5 submaximal isometric contractions as preconditioning for the tendon (Maganaris, 2003), the participant performs isometric ramped contractions with a gradual increase in force exertion from rest to maximum in about 5 seconds. The elongation of the patellar tendon during the contractions is visualized time-synchronized with the force or moment data using a linear ultrasound transducer overlying the tendon in the sagittal plane aligned with its longitudinal axis. Though the availability is currently limited, the use of a long linear ultrasound transducer (>6 cm) is to be recommended as it enables the visualization of the tendon origin and insertion at the patella apex and tibial tuberosity in one image (Mersmann et al., 2018). The displacement of the tendon insertion is currently tracked using self-developed manual tracking interfaces (e.g., Mersmann et al., 2017b) or (semi-)professional video analysis software (e.g., Image J^®^, Tracker^®^). Fully automated tracking might in near future replace these time-consuming procedures and enhance the objectivity of the analysis. To achieve a high reliability of the elongation measurement, three to five trials should be recorded and averaged (Schulze et al., 2012). The slope of a linear regression of the external force (or moment) and tendon elongation data between 50% and 100% of the exerted maximum force would be calculated as representative of tendon stiffness.

Due to its crucial importance for the estimation of injury risk, efficient muscle-tendon interaction and, thus, exercise prescription, an even more simplified assessment of tendon properties could be confined to tendon strain as outcome parameter. In such an approach, it would not be necessary to track the tendon insertion points over the full course of the contraction, yet only at rest and the plateau of the isometric maximum. In that case, the synchronization of ultrasound and force or moment data could be spared as well and tendon rest length and maximum elongation could theoretically be measured using the built-in software of the ultrasound device. Certainly, a validation and assessment of the reliability of the proposed procedures would be necessary and details still needs to be established how the approach can be most sensibly applied in different sports and environments in the future.

## Data Availability Statement

The raw data supporting the conclusions of this article will be made available by the authors, without undue reservation, to any qualified researcher.

## Ethics Statement

Ethics statements considering previously published work of our group can be found in the respective publications. Regarding the yet unpublished data, the participants (and legal guardians where applicable) gave written informed consent to the experimental procedures, which were approved by the Ethics Committee of the Humboldt-Universität zu Berlin (Ethikkommission der Kultur-, Sozial-, und Bildungswissenschaftlichen Fakultät; 16.02.2018) or the Ethics Committee of the Charité (Ethikausschuss 2 am Campus Virchow-Klinikum; ref. nr. EA2/076/15) and carried out in accordance with the Declaration of Helsinki.

## Author Contributions

AA, FM, and SB conceived the presented approach and drafted the manuscript. All authors approved the final version of the manuscript and agreed to be accountable for the content of the work.

## Conflict of Interest

The authors declare that the research was conducted in the absence of any commercial or financial relationships that could be construed as a potential conflict of interest.

## References

[B1] Albracht K. Arampatzis A. (2013). Exercise-induced changes in triceps surae tendon stiffness and muscle strength affect running economy in humans. *Eur. J. Appl. Physiol.* 113 1605–1615. 10.1007/s00421-012-2585-4 23328797

[B2] Arampatzis A. Karamanidis K. Albracht K. (2007a). Adaptational responses of the human Achilles tendon by modulation of the applied cyclic strain magnitude. *J. Exp. Biol.* 210 2743–2753. 10.1242/jeb.003814 17644689

[B3] Arampatzis A. Karamanidis K. Morey-Klapsing G. De Monte G. Stafilidis S. (2007b). Mechanical properties of the triceps surae tendon and aponeurosis in relation to intensity of sport activity. *J. Biomech.* 40 1946–1952. 10.1016/j.jbiomech.2006.09.005 17101142

[B4] Arampatzis A. Peper A. Bierbaum S. Albracht K. (2010). Plasticity of human Achilles tendon mechanical and morphological properties in response to cyclic strain. *J. Biomech.* 43 3073–3079. 10.1016/j.jbiomech.2010.08.014 20863501

[B5] Bashford G. R. Tomsen N. Arya S. Burnfield J. M. Kulig K. (2008). Tendinopathy discrimination by use of spatial frequency parameters in ultrasound B-mode images. *IEEE Trans. Med. Imaging* 27 608–615. 10.1109/TMI.2007.912389 18450534

[B6] Bohm S. Marzilger R. Mersmann F. Santuz A. Arampatzis A. (2018). Operating length and velocity of human vastus lateralis muscle during walking and running. *Sci. Rep.* 8:5066. 10.1038/s41598-018-23376-5 29567999 PMC5864755

[B7] Bohm S. Mersmann F. Arampatzis A. (2015). Human tendon adaptation in response to mechanical loading: a systematic review and meta-analysis of exercise intervention studies on healthy adults. *Sports Med. Open* 1:7. 10.1186/s40798-015-0009-9 PMC4532714 27747846

[B8] Bohm S. Mersmann F. Santuz A. Arampatzis A. (2019). The force–length–velocity potential of the human soleus muscle is related to the energetic cost of running. *Proc. R. Soc. Lond. B Biol. Sci.* 286:20192560. 10.1098/rspb.2019.2560 31847774 PMC6939913

[B9] Bohm S. Mersmann F. Tettke M. Kraft M. Arampatzis A. (2014). Human Achilles tendon plasticity in response to cyclic strain: effect of rate and duration. *J. Exp. Biol.* 217 4010–4017. 10.1242/jeb.112268 25267851

[B10] Burgess K. E. Connick M. J. Graham-Smith P. Pearson S. J. (2007). Plyometric vs. isometric training influences on tendon properties and muscle output. *J. Strength Cond. Res.* 21 986–989. 10.1519/R-20235.1 17685695

[B11] Carolan B. Cafarelli E. (1992). Adaptations in coactivation after isometric resistance training. *J. Appl. Physiol.* 73 911–917. 10.1152/jappl.1992.73.3.911 1400055

[B12] Charcharis G. Mersmann F. Bohm S. Arampatzis A. (2019). Morphological and mechanical properties of the quadriceps femoris muscle-tendon unit from adolescence to adulthood: effects of age and athletic training. *Front. Physiol.* 10:1082. 10.3389/fphys.2019.01082 31507446 PMC6718516

[B13] Cook J. L. Rio E. Purdam C. R. Docking S. I. (2016). Revisiting the continuum model of tendon pathology: what is its merit in clinical practice and research? *Br. J. Sports Med.* 50 1187–1191. 10.1136/bjsports-2015-095422 27127294 PMC5118437

[B14] Cormie P. McGuigan M. R. Newton R. U. (2011). Developing maximal neuromuscular power: part 1–biological basis of maximal power production. *Sports Med.* 41 17–38. 10.2165/11537690-000000000-00000 21142282

[B15] Couppé C. Svensson R. B. Josefsen C. O. Kjeldgaard E. Magnusson S. P. (2020). Ultrasound speckle tracking of Achilles tendon in individuals with unilateral tendinopathy: a pilot study. *Eur. J. Appl. Physiol.* 120 579–589. 10.1007/s00421-020-04317-5 32060739

[B16] Delecluse C. (1997). Influence of strength training on sprint running performance. *Sports Med.* 24 147–156. 10.2165/00007256-199724030-00001 9327528

[B17] Docking S. I. Cook J. (2019). How do tendons adapt? Going beyond tissue responses to understand positive adaptation and pathology development: a narrative review. *J. Musculoskelet. Neuronal Interact.* 19 300–310. 31475937 PMC6737558

[B18] Epro G. Hunter S. König M. Schade F. Karamanidis K. (2019). Evidence of a uniform muscle-tendon unit adaptation in healthy elite track and field jumpers: a cross sectional investigation. *Front. Physiol.* 10:574. 10.3389/fphys.2019.00574 31156457 PMC6529647

[B19] Epro G. Mierau A. Doerner J. Luetkens J. A. Scheef L. Kukuk G. M. (2017). The Achilles tendon is mechanosensitive in older adults: adaptations following 14 weeks versus 1.5 years of cyclic strain exercise. *J. Exp. Biol.* 220 1008–1018. 10.1242/jeb.146407 28298464

[B20] Fredberg U. Stengaard-Pedersen K. (2008). Chronic tendinopathy tissue pathology, pain mechanisms, and etiology with a special focus on inflammation. *Scand. J. Med. Sci. Sports* 18 3–15. 10.1111/j.1600-0838.2007.00746.x 18294189

[B21] Heinemeier K. M. Schjerling P. Heinemeier J. Magnusson S. P. Kjaer M. (2013). Lack of tissue renewal in human adult Achilles tendon is revealed by nuclear bomb (14)C. *FASEB J.* 27 2074–2079. 10.1096/fj.12-225599 23401563 PMC3633810

[B22] Hollville E. Nordez A. Guilhem G. Lecompte J. Rabita G. (2019). Interactions between fascicles and tendinous tissues in gastrocnemius medialis and vastus lateralis during drop landing. *Scand. J. Med. Sci. Sports* 29 55–70. 10.1111/sms.13308 30242912

[B23] Ishikawa M. Komi P. V. (2008). Muscle fascicle and tendon behavior during human locomotion revisited. *Exerc. Sport Sci. Rev.* 36 193–199. 10.1097/JES.0b013e3181878417 18815488

[B24] Kawakami Y. Fukunaga T. (2006). New insights into in vivo human skeletal muscle function. *Exerc. Sport Sci. Rev.* 34 16–21. 10.1097/00003677-200601000-00005 16394810

[B25] Kubo K. Morimoto M. Komuro T. Yata H. Tsunoda N. Kanehisa H. (2007). Effects of plyometric and weight training on muscle-tendon complex and jump performance. *Med. Sci. Sport Exerc.* 39 1801–1810. 10.1249/mss.0b013e31813e630a 17909408

[B26] Kurokawa S. Fukunaga T. Nagano A. Fukashiro S. (2003). Interaction between fascicles and tendinous structures during counter movement jumping investigated in vivo. *J. Appl. Physiol.* 95 2306–2314. 10.1152/japplphysiol.00219.2003 12871964

[B27] LaCroix A. S. Duenwald-Kuehl S. E. Lakes R. S. Vanderby R. (2013). Relationship between tendon stiffness and failure: a metaanalysis. *J. Appl. Physiol.* 115 43–51. 10.1152/japplphysiol.01449.2012 23599401 PMC3727010

[B28] Lai A. Lichtwark G. A. Schache A. G. Lin Y.-C. Brown N. A. T. Pandy M. G. (2015). In vivo behavior of the human soleus muscle with increasing walking and running speeds. *J. Appl. Physiol.* 118 1266–1275. 10.1152/japplphysiol.00128.2015 25814636

[B29] Lai A. Schache A. G. Lin Y.-C. Pandy M. G. (2014). Tendon elastic strain energy in the human ankle plantar-flexors and its role with increased running speed. *J. Exp. Biol.* 217 3159–3168. 10.1242/jeb.100826 24948642

[B30] Lai A. K. M. Lichtwark G. A. Schache A. G. Pandy M. G. (2018). Differences in in vivo muscle fascicle and tendinous tissue behavior between the ankle plantarflexors during running. *Scand. J. Med. Sci. Sports* 28 1828–1836. 10.1111/sms.13089 29603434

[B31] Lavagnino M. Arnoczky S. P. Kepich E. Caballero O. Haut R. C. (2008). A finite element model predicts the mechanotransduction response of tendon cells to cyclic tensile loading. *Biomech. Model. Mechanobiol.* 7 405–416. 10.1007/s10237-007-0104-z 17901992

[B32] Lichtwark G. A. Bougoulias K. Wilson A. M. (2007). Muscle fascicle and series elastic element length changes along the length of the human gastrocnemius during walking and running. *J. Biomech.* 40 157–164. 10.1016/j.jbiomech.2005.10.035 16364330

[B33] Lichtwark G. A. Wilson A. M. (2005). In vivo mechanical properties of the human Achilles tendon during one-legged hopping. *J. Exp. Biol.* 208 4715–4725. 10.1242/jeb.01950 16326953

[B34] Lichtwark G. A. Wilson A. M. (2007). Is Achilles tendon compliance optimised for maximum muscle efficiency during locomotion? *J. Biomech.* 40 1768–1775. 10.1016/j.jbiomech.2006.07.025 17101140

[B35] Maganaris C. N. (2003). Tendon conditioning: artefact or property? *Proc. R. Soc. B Biol. Sci.* 270 S39–S42. 10.1098/rsbl.2003.0004 12952631 PMC1698017

[B36] Magnusson S. P. Langberg H. Kjaer M. (2010). The pathogenesis of tendinopathy: balancing the response to loading. *Nat. Rev. Rheumatol.* 6 262–268. 10.1038/nrrheum.2010.43 20308995

[B37] Masterson D. W. (1976). The ancient Greek origins of sports medicine. *Br. J. Sports Med.* 10 196–202. 10.1136/bjsm.10.4.196 795492 PMC1859519

[B38] McCrum C. Leow P. Epro G. König M. Meijer K. Karamanidis K. (2018). Alterations in leg extensor muscle-tendon unit biomechanical properties with ageing and mechanical loading. *Front. Physiol.* 9:2743. 10.3389/fphys.2018.00150 29541035 PMC5835978

[B39] McMaster D. T. Gill N. Cronin J. McGuigan M. (2014). A brief review of strength and ballistic assessment methodologies in sport. *Sports Med.* 44 603–623. 10.1007/s40279-014-0145-2 24497158

[B40] Mersmann F. Bohm S. Arampatzis A. (2017a). Imbalances in the development of muscle and tendon as risk factor for tendinopathies in youth athletes: a review of current evidence and concepts of prevention. *Front. Physiol.* 8:987. 10.3389/fphys.2017.00987 29249987 PMC5717808

[B41] Mersmann F. Bohm S. Schroll A. Marzilger R. Arampatzis A. (2016). Athletic training affects the uniformity of muscle and tendon adaptation during adolescence. *J. Appl. Physiol.* 121 893–899. 10.1152/japplphysiol.00493.2016 27586836

[B42] Mersmann F. Charcharis G. Bohm S. Arampatzis A. (2017b). Muscle and tendon adaptation in adolescence: elite volleyball athletes compared to untrained boys and girls. *Front. Physiol.* 8:613. 10.3389/fphys.2017.00417 28670285 PMC5472702

[B43] Mersmann F. Pentidis N. Tsai M. S. Schroll A. Arampatzis A. (2019). Patellar tendon strain associates to tendon structural abnormalities in adolescent athletes. *Front. Physiol.* 10:963. 10.3389/fphys.2019.00963 31427983 PMC6687848

[B44] Mersmann F. Seynnes O. R. Legerlotz K. Arampatzis A. (2018). Effects of tracking landmarks and tibial point of resistive force application on the assessment of patellar tendon mechanical properties in vivo. *J. Biomech.* 71 176–182. 10.1016/j.jbiomech.2018.02.005 29463386

[B45] Mitchell C. J. Churchward-Venne T. A. West D. W. D. Burd N. A. Breen L. Baker S. K. (2012). Resistance exercise load does not determine training-mediated hypertrophic gains in young men. *J. Appl. Physiol*. 113, 71–77. 10.1152/japplphysiol.00307.2012 22518835 PMC3404827

[B46] Nikolaidou M. E. Marzilger R. Bohm S. Mersmann F. Arampatzis A. (2017). Operating length and velocity of human M. vastus lateralis fascicles during vertical jumping. *R. Soc. Open Sci.* 4:170185. 10.1098/rsos.170185 28573027 PMC5451828

[B47] Orselli M. I. V. Franz J. R. Thelen D. G. (2017). The effects of Achilles tendon compliance on triceps surae mechanics and energetics in walking. *J. Biomech.* 60 227–231. 10.1016/j.jbiomech.2017.06.022 28728791 PMC5555172

[B48] Osternig L. R. (1986). Isokinetic dynamometry: implications for muscle testing and rehabilitation. *Exerc. Sport Sci. Rev.* 14 45–80. 3525192

[B49] Pentidis N. Mersmann F. Bohm S. Giannakou E. Aggelousis N. Arampatzis A. (2019). Triceps surae muscle-tendon unit properties in preadolescent children: a comparison of artistic gymnastic athletes and non-athletes. *Front. Physiol.* 10:615. 10.3389/fphys.2019.00615 31164838 PMC6536691

[B50] Pizzolato C. Lloyd D. G. Zheng M. H. Besier T. F. Shim V. B. Obst S. J. (2018). Finding the sweet spot via personalised Achilles tendon training: the future is within reach. *Br. J. Sports Med.* 53 11–12. 10.1136/bjsports-2018-099020 30030281

[B51] Raglin J. S. (2001). Psychological factors in sport performance: the mental health model revisited. *Sports Med.* 31 875–890. 10.2165/00007256-200131120-00004 11665914

[B52] Roberts T. J. (1997). Muscular force in running Turkeys: the economy of minimizing work. *Science* 275 1113–1115. 10.1126/science.275.5303.1113 9027309

[B53] Roberts T. J. (2016). Contribution of elastic tissues to the mechanics and energetics of muscle function during movement. *J. Exp. Biol.* 219 266–275. 10.1242/jeb.124446 26792339 PMC6514471

[B54] Robinson P. (2009). Sonography of common tendon injuries. *Am. J. Roentgenol.* 193 607–618. 10.2214/AJR.09.2808 19696272

[B55] Sáez-Sáez de Villarreal E. Requena B. Newton R. U. (2010). Does plyometric training improve strength performance? A meta-analysis. *J. Sci. Med. Sport* 13 513–522. 10.1016/j.jsams.2009.08.005 19897415

[B56] Sawicki G. S. Robertson B. D. Azizi E. Roberts T. J. (2015). Timing matters: tuning the mechanics of a muscle–tendon unit by adjusting stimulation phase during cyclic contractions. *J. Exp. Biol.* 218 3150–3159. 10.1242/jeb.121673 26232413 PMC4631775

[B57] Schoenfeld B. J. (2013). Potential mechanisms for a role of metabolic stress in hypertrophic adaptations to resistance training. *Sports Med.* 43 179–194. 10.1007/s40279-013-0017-1 23338987

[B58] Schulze F. Mersmann F. Bohm S. Arampatzis A. (2012). A wide number of trials is required to achieve acceptable reliability for measurement patellar tendon elongation in vivo. *Gait Posture* 35 334–338. 10.1016/j.gaitpost.2011.09.107 22178032

[B59] Seynnes O. R. Bojsen-Moller J. Albracht K. Arndt A. Cronin N. J. Finni T. (2015). Ultrasound-based testing of tendon mechanical properties: a critical evaluation. *J. Appl. Physiol.* 118 133–141. 10.1152/japplphysiol.00849.2014 25414247

[B60] Smith D. J. Norris S. R. Hogg J. M. (2002). Performance evaluation of swimmers: scientific tools. *Sports Med.* 32 539–554. 10.2165/00007256-200232090-00001 12096928

[B61] Stafilidis S. Arampatzis A. (2007). Muscle – tendon unit mechanical and morphological properties and sprint performance. *J. Sports Sci.* 25 1035–1046. 10.1080/02640410600951589 17497405

[B62] Stenroth L. Peltonen J. Cronin N. J. Sipila S. Finni T. (2012). Age-related differences in Achilles tendon properties and triceps surae muscle architecture in vivo. *J. Appl. Physiol.* 113 1537–1544. 10.1152/japplphysiol.00782.2012 23042907

[B63] Suchomel T. J. Nimphius S. Stone M. H. (2016). The Importance of muscular strength in athletic performance. *Sports Med.* 46 1419–1449. 10.1007/s40279-016-0486-0 26838985

[B64] van Schie H. T. M. de Vos R. J. de Jonge S. Bakker E. M. Heijboer M. P. Verhaar J. A. N. (2010). Ultrasonographic tissue characterisation of human Achilles tendons: quantification of tendon structure through a novel non-invasive approach. *Br. J. Sports Med.* 44 1153–1159. 10.1136/bjsm.2009.061010 19666626

[B65] Visnes H. Tegnander A. Bahr R. (2015). Ultrasound characteristics of the patellar and quadriceps tendons among young elite athletes. *Scand. J. Med. Sci. Sports* 25 205–215. 10.1111/sms.12191 24612006

[B66] Wang T. Lin Z. Day R. E. Gardiner B. Landao-Bassonga E. Rubenson J. (2013). Programmable mechanical stimulation influences tendon homeostasis in a bioreactor system. *Biotechnol. Bioeng.* 110 1495–1507. 10.1002/bit.24809 23242991

[B67] Waugh C. M. Blazevich A. J. Fath F. Korff T. (2012). Age-related changes in mechanical properties of the Achilles tendon. *J. Anat.* 220 144–155. 10.1111/j.1469-7580.2011.01461.x 22150089 PMC3275769

[B68] Waugh C. M. Korff T. Fath F. Blazevich A. J. (2014). Effects of resistance training on tendon mechanical properties and rapid force production in prepubertal children. *J. Appl. Physiol.* 117 257–266. 10.1152/japplphysiol.00325.2014 24903920 PMC4122689

[B69] Wiesinger H.-P. Kösters A. Müller E. Seynnes O. R. (2015). Effects of Increased Loading on in vivo tendon properties: a systematic review. *Med. Sci. Sport Exerc.* 47 1885–1895. 10.1249/MSS.0000000000000603 25563908 PMC4535734

[B70] Wren T. A. L. Lindsey D. P. Beaupré G. S. Carter D. R. (2003). Effects of creep and cyclic loading on the mechanical properties and failure of human Achilles tendons. *Ann. Biomed. Eng.* 31 710–717. 10.1114/1.1569267 12797621

[B71] Yarrow K. Brown P. Krakauer J. W. (2009). Inside the brain of an elite athlete: the neural processes that support high achievement in sports. *Nat. Rev. Neurosci.* 10 585–596. 10.1038/nrn2672 19571792

